# Spinal SIRT1 Activation Attenuates Neuropathic Pain in Mice

**DOI:** 10.1371/journal.pone.0100938

**Published:** 2014-06-24

**Authors:** Haijun Shao, Qingsheng Xue, Fujun Zhang, Yan Luo, Hao Zhu, Xiaoqing Zhang, Honghai Zhang, Wenlong Ding, Buwei Yu

**Affiliations:** 1 Department of Anesthesiology, Ruijin Hospital, School of Medicine, Shanghai Jiao Tong University, Shanghai, PR China; 2 Department of Anatomy, School of Medicine, Shanghai Jiao Tong University, Shanghai, PR China; 3 Department of Anesthesiology, Hangzhou First People's Hospital, Nanjing Medical University, Zhejiang, PR China; University of Texas Medical Branch, United States of America

## Abstract

Abnormal histone acetylation occurs during neuropathic pain through an epigenetic mechanism. Silent information regulator 1 (sir2 or SIRT1), a NAD-dependent deacetylase, plays complex systemic roles in a variety of processes through deacetylating acetylated histone and other specific substrates. But the role of SIRT1 in neuropathic pain is not well established yet. The present study was intended to detect SIRT1 content and activity, nicotinamide (NAM) and nicotinamide adenine dinucleotide (NAD) in the spinal cord using immunoblotting or mass spectroscopy over time in mice following chronic constriction injury (CCI) or sham surgery. In addition, the effect of intrathecal injection of NAD or resveratrol on thermal hyperalgesia and mechanical allodynia was evaluated in CCI mice. Finally, we investigated whether SIRT1 inhibitor EX-527 could reverse the anti-nociceptive effect of NAD or resveratrol. It was found that spinal SIRT1 expression, deacetylase activity and NAD/NAM decreased significantly 1, 3, 7, 14 and 21 days after CCI surgery as compared with sham group. In addition, daily intrathecal injection of 5 µl 800 mM NAD 1 h before and 1 day after CCI surgery or single intrathecal injection of 5 µl 90 mM resveratrol 1 h before CCI surgery produced a transient inhibitory effect on thermal hyperalgesia and mechanical allodynia in CCI mice. Finally, an intrathecal injection of 5 µl 1.2 mM EX-527 1 h before NAD or resveratrol administration reversed the anti-nociceptive effect of NAD or resveratrol. These data indicate that the reduction in SIRT1 deacetylase activity may be a factor contributing to the development of neuropathic pain in CCI mice. Our findings suggest that the enhancement of spinal NAD/NAM and/or SIRT1 activity may be a potentially promising strategy for the prevention or treatment of neuropathic pain.

## Introduction

Neuropathic pain is characterized by both negative symptoms and positive symptoms including hyperalgesia, allodynia, paresthesia and spontaneous pain [1]. Many studies [2], [3], [4], [5] suggest that the symptoms of neuropathic pain can be attributed to a variety of alterations in pain-related gene expression and modification in primary afferent or spinal cord neurons. One of the characteristic alterations in gene modification is abnormal histone acetylation, which is believed to be one of the transcription factor-mediated epigenetic mechanisms underlying neuropathic pain [6], [7]. Several classes of histone deacetylases (HDACs) are expressed in the spinal cord that is a critical structure of the nociceptive pathway [8], [9]. Silent information regulator (SIRT) can act as a HDAC deacetylating acetylated histone.

Sir2 or SIRT1, a member of SIRT family, is a NAD-dependent deacetylase. It was initially identified as a mediator of longevity in yeast [10]. Its mammalian homologue, SIRT1, is a class III HDAC and plays important roles in many physiopathological conditions such as diabetes, cardiovascular disorders, cancer and neurodegeneration [11], [12], [13], [14]. The specificity of SIRT1 catalytic activity relies on two domains of the substrate binding site: a Rossmann -like domain that binds to NAD [15], and an alpha-helix domain with a zinc ribbon for zinc binding. SIRT1 deacetylation needs NAD as a cofactor, which is consumed by SIRT1 and then into NAM [16]. In addition, NAM inhibits genome silencing and accelerates aging by regulating yeast sir2 at 50–150 µM concentrations, and maintaining the NAD/NAM ratio may be crucial for the normal SIRT1 catalytic activity [17]. In addition, SIRT1 is a major effector of axonal protection mediated by increased NAD biosynthesis [18]. Resveratrol (3,4′,5-trihydroxystilbene), a phytoalexin naturally present in plants, binds to SIRT1 at the N-terminal and then increases SIRT1 activity [19], showing promising potential for the treatment of pain mediated by nucleus pulposus [20] and the attenuation of neuropathic pain [21], [22] and inflammatory pain [23]. EX-527 (6-chloro-2, 3, 4, 9-tetrahydro-1-H-carbazole-1-carboxamide), a SIRT1 inhibitor that is more potent and selective than other current SIRT1 inhibitors [24], can induce p53 acetylation and cell death through targeting SIRT1 [25]. Recently, SIRT1 has been implicated in the pathologic process in lumbar segment of the spinal cord in a SOD1^G93A^ transgenic mouse model of amyotrophic lateral sclerosis [26], suggesting that SIRT1 may play some role in pain modulation. However, the role of SIRT1 in neuropathic pain is not well established yet.

We therefore hypothesized that the decreased SIRT1 deacetylase activity may be a contributing factor of the development of neuropathic pain. We set out to observe the SIRT1 content and activity, NAM and NAD in the spinal cord using immunoblotting or mass spectroscopy over time in mice following chronic constriction injury (CCI) or sham surgery. The effect of intrathecal injection of NAD or resveratrol on thermal hyperalgesia and mechanical allodynia in CCI mice was then evaluated. Finally, we investigated whether SIRT1 inhibitor EX-527 could reverse the anti-nociceptive effect of NAD or resveratrol. It was found that CCI surgery decreased SIRT1expression/activity and the NAD/NAM ratio in mice. The intrathecal injection of 5 µl 800 mM NAD or 90 mM resveratrol might be effective to modulate the CCI-induced development of neuropathic pain by promoting NAD-dependent SIRT1 deacetylase activity. In addition, an intrathecal injection of 5 µl 1.2 mM EX-527 1 h before the NAD or resveratrol administration reversed the anti-nociceptive effect of increased spinal NAD or exogenous resveratrol, suggesting that the SIRT1 activation may be essential for the effect of NAD or resveratrol. Our data suggest that the enhancement of SIRT1 activity may be a potentially promising pharmacological strategy for the prevention or treatment of neuropathic pain.

## Materials and Methods

### Subjects

All experimental protocols were approved by the Animal Care and Use Committee of Shanghai JiaoTong University School of Medicine (Shanghai, China). All surgical procedures were performed under general anaesthesia, and every effort was made to minimize stress. Adult male Kunming mice (*n* = 384) weighing 20–25 g (SLAC Laboratory Animal Co., Ltd., Shanghai, China) were housed in a temperature-controlled (22°C) colony room under a 12 h/12 h light/dark cycle regime, with food and water available *ad libitum*. All behavioral testing was performed during the light cycle between 10∶00 AM and 3∶00 PM. The mice were randomly allocated to drug- or vehicle-treated groups. Each behavioral test group included 10 or 12 mice and each molecular detection group included 6 mice.

### Intrathecal injection

Drugs or vehicles were intrathecally injected into mice as the method described by Hylden et al [27]. Briefly, a 30 gauge stainless steel needle attached to a 10-µl Hamilton microsyringe was inserted at an angle about 20° above the vertebral column into L4 or L5 spinous space, and then advanced carefully to the intervertebral space as the angle of the syringe was decreased to about 10° until a sudden slight flick of the tail was observed, indicating the tip of the needle had entered the subarachnoid space. The drug or the vehicle was then injected in a 5 µl volume over a 30 s period into the subarachnoid space and the needle was left in place for further 15 s before withdrawl. NAD and resveratrol were purchased from Sigma (St. Louis, MO) and EX-527 was a gift from Lei Zhang (the Ninth People's Hospital, Shanghai, China). The doses of NAD, resveratrol and EX-527 were selected according to previous reports and our preliminary experiments. Specifically, 400 mM and 800 mM NAD was prepared in saline [18], [28], [29], 45 mM and 90 mM resveratrol was dissolved in 20% DMSO [21], [30], [31] and 1.2 mM EX-527 was prepared in 20% DMSO [24]. The dosing schedule of drugs or vehicles was present in the Results or Figures. Motor functions were cursorily evaluated by observing the placing/stepping reflexes and righting reflex 5 min before the next nociceptive test or drug administration. Animals with signs of motor impairment were excluded from the experiment.

### Neuropathic pain model

After a pre-evaluation of pain, mice received either chronic constriction injury of the sciatic nerve or sham surgery by only exposing the sciatic nerve without ligation as described previously [32]. Briefly, the left sciatic nerve was exposed at the mid-thigh level under sodium pentobarbital anesthesia (65 mg kg^−1^, i.p.). Three ligations (4–0 chromic gut, with a distance about 1 mm apart) were loosely constricted around the nerve proximal to the trifurcation by blunt dissection through the biceps femoris. The tension of the constriction was guided by the occurrence of a short flick of the ipsilateral hind limb.

### Assessment of thermal hyperalgesia and mechanical allodynia

The investigator was unaware of the drug or vehicle administration that the mouse had received. Thermal hyperalgesia was evaluated by radiant heat paw withdrawal test as described by Hargreaves et al [33]. Briefly, the mouse was individually placed on a 1 mm thick and smooth glass surface surrounded by a plastic chamber (7×9×11 cm) and the heat source was provided by an analgesia meter located below the glass surface. The mouse was placed in the chamber to acclimatize to the environment for 45 min before behavior tests. The heat source was focused on a portion of the ipsilateral hind paws of the mouse and an automatic 20-s cutoff was used to prevent tissue damage. Each hind paw was tested three times with a of 5-min interval between two measurements to avoid habituation. Mechanical allodynia was determined by observing the occurrence of foot withdrawal in response to mechanical indentation of the plantar surface of each hind paw with Von Frey filaments (North Coast Medical Inc., CA, USA). Before starting the experiments, we tested the actual forces of the series of 8 von Frey filaments (actual force: 0.31, 0.38, 0.45, 0.6, 1.0, 1.4, 2.0, and 4.0 g). The mouse was placed individually in a 20×25×15 cm plastic box on a wire mesh floor and then allowed to acclimate for 45 min. The filaments were perpendicularly presented to 10 designated loci distributed over the plantar surface of the hind paws with a force to cause slight bending against the hind paw, and held for 4–5 s. The filaments were applied in order of ascending force, starting with 0.31 g and ending with 4.0 g as cutoff value with a 10–15 s interval. Each filament was applied alternately to each foot and locus. The incidence of foot withdrawal was expressed as the number of positive responses multiplied by 10 and the percentage of response of each filament was then plotted as a function of force. For each mouse, ascending forces were tested either until the maximum stimulus (4.0 g) was reached or until a hair force was achieved that caused 10 positive responses. The mechanical allodynia was defined as the force that produced a minimum detectable withdrawal observed on 50% of the tests at the same force level. In cases where none of the specific filaments exactly produced 5 positive withdrawals in 10 tests, the linear interpolation was calculated as the threshold.

### Immunoblotting

The animals were sacrificed by overdose urethane after the supposed survival time. The L4–5 spinal cord was dissected and removed, and the dorsal horn of the left half of L4 and L5 was isolated and then frozen immediately in liquid nitrogen. Tissue samples were homogenized in lysis buffer (12.5 µl mg^−1^ tissue) containing (in mM) Tris 20.0, sucrose 250.0, Na_3_VO_4_ 0.03, MgCl_2_ 2.0, EDTA 2.0, EGTA 2.0, phenylmethylsulfonyl fluoride 2.0, dithiothreitol (DTT) 1.0, and protease inhibitor cocktail 0.02% (v/v), pH 7.4. The homogenate was centrifuged at 13,000 rpm for 15 min at 4°C and the supernatant was collected for protein concentration determination by using Bradford method [34]. Primary antibody SIRT1 (1∶2000; Abcam, Cambridge, UK), H4-k16Ac (1∶2000) and H4 (1∶1000) (Cell Signaling Technology, Danvers, Massachusetts) were used to detect SIRT1, H4-16Ac and H4.

### Assessment of NAD and NAM

An aliquot of the frozen spinal cord was lysed in 200 µl HClO_4_ (1.0 M) containing 3.2 nmol [^18^O] NAD and 1.4 nmol [^18^O] NAM. The supernatant was collected after centrifugation at 12,000 rpm for 10 min at RT, neutralized with NaOH and injected on a C-18 semipreparative column. NAD and NAM fractions were collected according to the retention time of authentic standards, lyophilized and redissolved in 50% acetonitrile. Matrix-assisted laser desorption ionization-mass spectroscopy (MS) (positive mode) was used to detect NAD, and ESI-MS (positive mode) was used to measure NAM signal. Peak areas for NAD were determined by integration of total ion at mass 646 amu and at 666 amu, and the peak was calculated with the formula: ^16^O pmol  =  (646 ion area/666 ion area –0.035) ×^18^O pmol. The peak ratio of 123/125 provided quantity of the nmol of NAM. ^16^O pmol  =  (123 ion area/125 ion area –0.035) ×^18^O pmol. The NAD or NAM concentrations in tissue were obtained from NAD and NAM contents divided by the tissue volume (density, 1.0 µl mg^−1^ of tissue). Appropriate blanks and controls were also analyzed together.

### Statistical Analysis

Data were presented as means ± SEM. Comparisons in withdrawal latencies over time were performed by using two-way analysis of variance (ANOVA) with repeated measures followed by Bonferroni post hoc tests. Western blot and MS data between the groups were analyzed by student's *t* test or one-way analysis of variance. The results of all analyses were considered significant at *P*<0.05.

## Results

### Down-regulation of SIRT1 and NAD in the spinal cord of CCI mice

**Figure 1:**
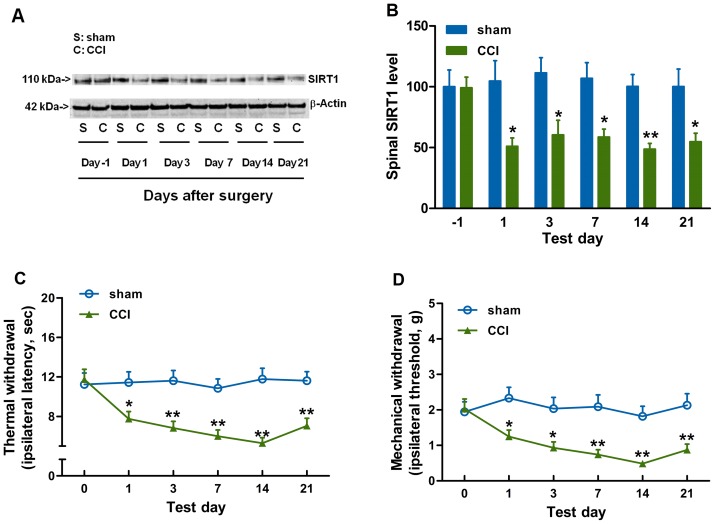
CCI surgery decreases spinal SIRT1 content and this is associated with an increase in pain-related behaviors. (A) Spinal SIRT1 content is decreased 1, 3, 7, 14 and 21 days after CCI surgery as compared with that after sham surgery. (B) Quantification of the immunoblots shows that SIRT1 levels are decreased after CCI surgery. CCI surgery induces (C) thermal hyperalgesia and (D) mechanical allodynia in ipsolateral hind paw. *n* = 12 in each group for behavioral test and *n* = 6 at each time point in each group for SIRT1 analysis; ^*^ *P*<0.05, ^**^ *P*<0.01 *vs.* sham group at each time point. CCI, chronic constriction injury; SIRT1, silent information regulator 1.

**Figure 2:**
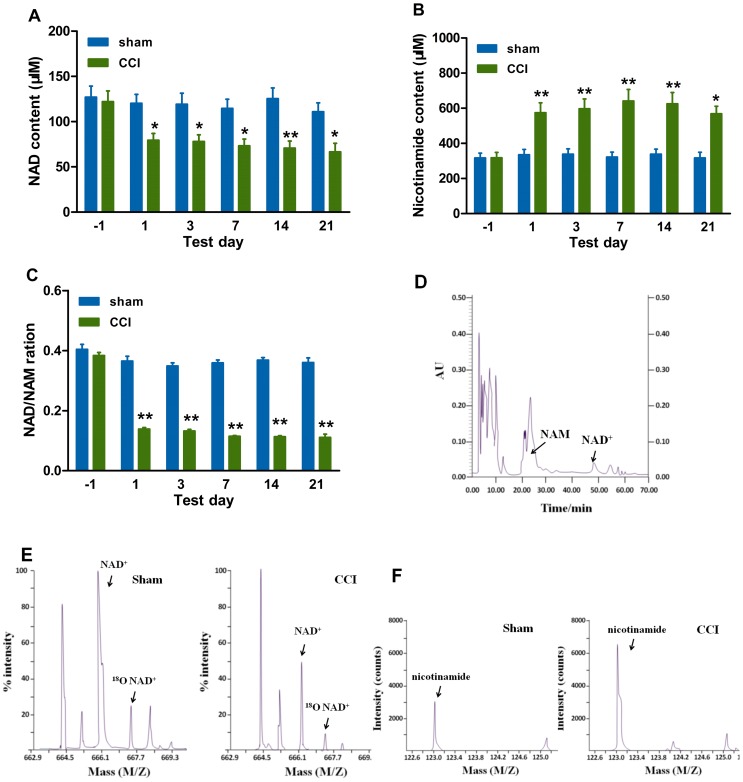
CCI surgery decreases NAD and increases NAM in the spinal cord. CCI surgery decreases (A) NAD and increases (B) NAM as compared with sham surgery. (C) The ratio of NAD/NAM is decreased in CCI mice as compared with that in sham group. (D) NAM and NAD fractions are collected according to HPLC method. The arrows show the retention time of NAM and NAD, which is at around 21 min and 48 min, respectively. The NAM peaks are mixed with those of other impurities. (E) Spectrograms of NAD with MALDI-MS are representative results from a sham-treated (left) and a CCI-treated (right) mouse. The position of NAD and ^18^O NAD are indicated. (F) Spectrograms of NAM with ESI-MS are representative results from a sham-treated (left) and a CCI-treated (right) mouse. The position of NAM is indicated. *n* = 6 at each time point in each group; ^*^ *P*<0.05, ^**^ *P*<0.01 *vs.* sham group at each time point. NAM, nicotinamide; NAD, nicotinamide adenine dinucleotide; CCI, chronic constriction injury; MALDI-MS, matrix-assisted laser desorption ionization-mass spectroscopy; ESI-MS, electrospray ionization-mass spectrometry.

**Figure 3:**
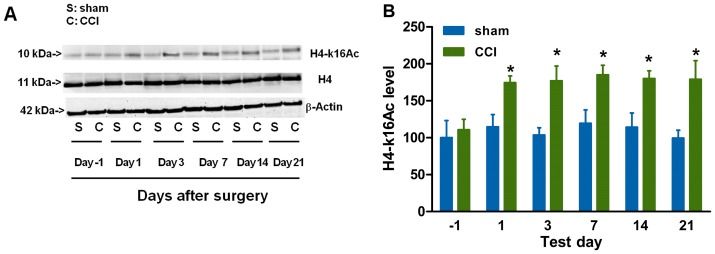
CCI surgery increases acetylation of H4-k16. (A) The levels of H4-k16Ac, but not total H4, are increased 1, 3, 7, 14 and 21 days after CCI surgery as compared with those after sham surgery. (B) Quantification of the immunoblots shows that H4-k16Ac is increased after CCI surgery. *n* = 6 at each time point in each group; ^*^ *P*<0.05, ^**^ *P*<0.01 *vs.* sham group at each time point. CCI, chronic constriction injury; SIRT1, silent information regulator 1; H4-k16Ac, H4-k16 acetylation.

The decreased SIRT1 expression or activity in CCI-induced neuropathic pain is a prerequisite of our hypothesis. Mice were sacrificed for molecular detection before, and at 1, 3, 7, 14 and 21 days after CCI or sham surgery. It was found that CCI surgery induced a decrease in SIRT1 protein content (Fig. 1A and 1B) in the spinal cord, accompanied with an increase in thermal hyperalgesia (Fig. 1C) and mechanical allodynia (Fig. 1D), as compared with sham group. However, sham surgery had no significant effect on SIRT1 content (Fig. 1A and 1B). Knowing that SIRT1 consumes NAD and then releases NAM, we further investigated the effect of CCI surgery on NAD and NAM content. The content of NAD was decreased (Fig. 2A) and the content of NAM was increased (Fig. 2B) in CCI mice as compared with those in sham group. Thus, the NAD/NAM ratio (Fig. 2C) was decreased in CCI mice as compared with that in sham group, which was equal to the aberration of SIRT1 content. NAM and NAD fractions were collected with HPLC method at around 21 min and 48 min, respectively (Fig. 2D). Then, NAD and NAM were detected with MALDI-MS (Fig. 2E) and ESI-MS (Fig. 2F), respectively. The decrease in SIRT1 protein content and NAD/NAM ratio in the spinal cord of CCI mice coincided with a marked reduction in the SIRT1 deacetylase activity as represented by an apparent elevation in acetylation level of SIRT1 substrate H4-k16Ac (acetylation of H4-k16) (Fig. 3A and 3B) in the spinal cord, as determined by immunoblots using an antibody specific for H4-k16Ac residue [35]. Similarly, no significant effect of sham surgery on SIRT1 deacetylase activity was observed (Fig. 3A and 3B), suggesting that CCI surgery, but not exposure of the sciatic nerve, decreased the SIRT1 activity. One of the possible explanations for the observed elevation of immunoreactive H4-k16Ac content was an increase in total H4 content. In contrast, the total H4 content in the spinal cord did not undergo significant change in CCI mice as compared with sham group (Fig. 3A), indicating that the increase in immunoreactive H4-K16Ac content in the spinal cord was attributed to the reduction in deacetylation. Accordingly, we did not further detect total H4 protein content in the subsequent parts of the study. Additionally, H3-k9Ac (acetylation of H3-k9) levels were also increased in CCI mice relative to those before CCI surgery (data not shown). Our data were consistent with other studies demonstrating that SIRT1 deacetylated histone polypeptides with a preference for H4-k16Ac and H3-K9Ac [35]. In this study, H4-k16Ac signaling was used as the specific indicator for SIRT1-mediated deacetylase activity. Taken together, CCI surgery decreases SIRT1 content/activity and NAD/NAM in the mouse spinal cord.

### NAD attenuates the CCI-induced development of neuropathic pain

**Figure 4:**
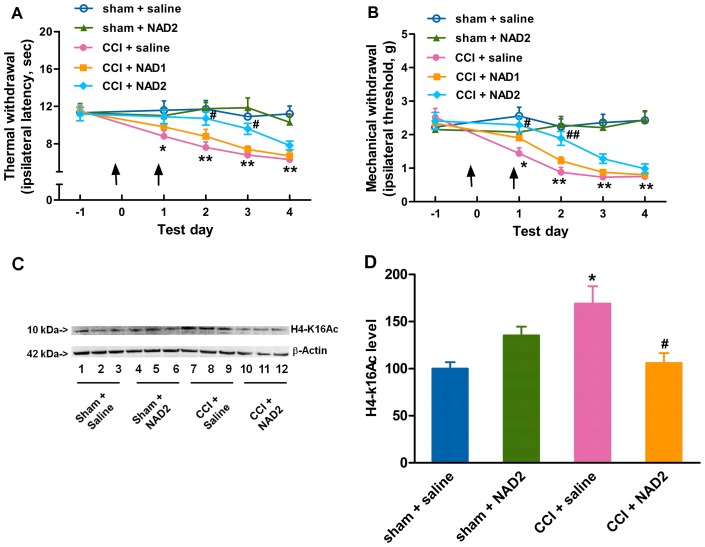
NAD attenuates the development of neuropathic pain by alleviating the reduction of SIRT1 deacetylase activity. Daily intrathecal injections of 5 µl 800 mM NAD into mice 1 h before and 1 day after CCI surgery, but not 400 mM, produce transient inhibition of the development of (A) thermal hyperalgesia and (B) mechanical allodynia induced by CCI surgery. (C) 800 mM NAD attenuates CCI-induced elevation of H4-K16Ac 2 days after CCI surgery. (D) Quantification of the immunoblots shows that 800 mM NAD attenuates CCI-induced increase in H4-K16Ac. An arrow indicates an intrathecal injection. *n* = 10 in each group for behavior test and *n* = 6 in each group for H4-K16Ac analysis; ^*^ *P*<0.05, ^**^ *P*<0.01 *vs.* saline-treated sham group; ^#^ *P*<0.05, ^##^ *P*<0.01 *vs.* saline-treated CCI group. NAD1, 400 µM nicotinamide adenine dinucleotide; NAD2, 800 µM nicotinamide adenine dinucleotide; CCI, chronic constriction injury; H4-k16Ac, H4-k16 acetylation.

Neuronal cells express membrane proteins which can bind and transport extracellular NAD into the cell [36]. Therefore, we investigated whether the exogenous NAD could attenuate the CCI-induced neuropathic pain. Daily intrathecal injection of 5 µl 800 mM NAD [28], [29] into mice 1 h before and 1 day after CCI surgery, but not 400 mM, produced transient inhibition on the development of thermal hyperalgesia (Fig. 4A) and mechanical allodynia (Fig. 4B), with the degree of pain returning within 48 h after the last injection, as compared with the saline-treated CCI group. Additionally, 800 mM NAD attenuated the CCI-induced elevation of H4-k16Ac (Fig. 4C and 4D) 2 days after CCI surgery in mice as compared with the saline-treated CCI group. Taking into account that the levels of H3 or H4 acetylation can be used to evaluate the deacetylase activity of SIRT1 [35], our data suggested that abundant exogenous NAD injected intrathecally attenuated the development of neuropathic pain through an epigenetic mechanism in the mouse spinal cord.

### Resveratrol attenuates the CCI-induced development of neuropathic pain

**Figure 5:**
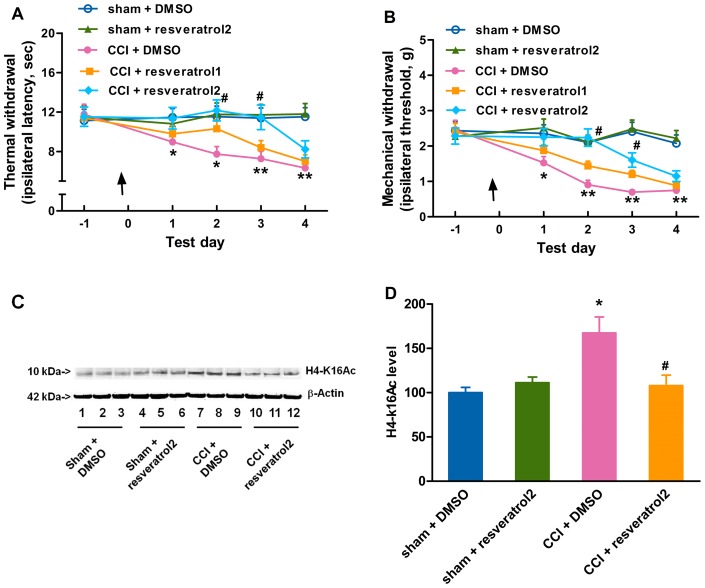
Resveratrol attenuates the development of neuropathic pain by alleviating the reduction of SIRT1 deacetylase activity. An intrathecal administration of 5 µl 90 mM resveratrol 1 h before CCI surgery, but not 45 mM suppresses the development of (A) thermal hyperalgesia and (B) mechanical allodynia induced by CCI surgery. (C) 45 mM resveratrol attenuates CCI-induced elevation of H4-K16Ac 2 days after CCI surgery. (D) Quantification of the immunoblots shows that 45 mM resveratrol attenuates CCI-induced increase in H4-K16Ac 2 days after surgery. An arrow indicates an intrathecal injection. *n* = 10 in each group for behavior test and *n* = 6 in each group for H4-K16Ac analysis; ^*^ *P*<0.05, ^**^ *P*<0.01 *vs.* DMSO-treated sham group; ^#^ *P*<0.05, ^##^ *P*<0.01 *vs.* DMSO-treated CCI group. Resveratrol1, 45 µM resveratrol; resveratrol2, 90 µM resveratrol; CCI, chronic constriction injury; H4-k16Ac, H4-k16 acetylation.

Knowing that resveratrol has an anti-nociceptive effect in some pain models [20], [21], [22], [23] and works as an activator of SIRT1 as reported in the literature [37], [38], we further asked whether resveratrol could delay the development of neuropathic pain. It was found in our study that an intrathecal administration of 5 µl 90 mM resveratrol [21], [30], [31] 1 h before CCI surgery, but not 45 mM, delayed the onset of thermal hyperalgesia (Fig. 5A) and mechanical allodynia (Fig. 5B), as comparable to the effect of exogenous NAD (Fig. 4A and 4B). Meanwhile, 90 mM resveratrol injected intrathecally reversed the CCI-induced elevation of H4-k16Ac (Fig. 5C and 5D) 2 days after CCI surgery in mice, as compared with the DMSO-treated CCI group. However, resveratrol had no significant effects on sham-operated mice. These results tempted us to conclude that resveratrol may delay the development of neuropathic pain induced by CCI and the reduction of SIRT1 activity may be one of the responsible factors contributing to the CCI-induced neuropathic pain.

### Normal SIRT1 activity is essential for the anti-nociceptive effect of NAD and resveratrol

**Figure 6:**
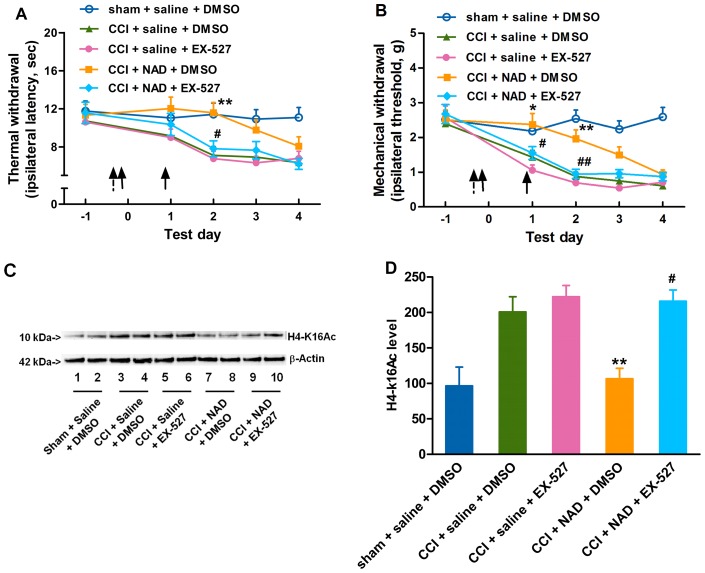
EX-527 inhibits the anti-nociceptive effect of NAD. An intrathecal injection of 5 µl EX-527 (1.2 mM) 1 h before NAD administration effectively reverses the effect of NAD (800 mM) delaying the onset of (A) thermal hyperalgesia and (B) mechanical allodynia produced by CCI surgery. (C) EX-527 inhibits the effect of NAD attenuating CCI-induced elevation of H4-K16Ac 1 day after CCI surgery. (D) Quantification of the immunoblots shows that EX-527 inhibits the effects of NAD on H4-K16Ac. An arrow indicates a NAD injection and a dotted arrow indicates an EX-527 injection. *n* = 10 in each group for behavior test and *n* = 6 in each group for H4-K16Ac analysis; ^*^ *P*<0.05, ^**^ *P*<0.01 *vs.* saline-DMSO-treated CCI group; ^#^ *P*<0.05, ^##^ *P*<0.01 *vs.* NAD-DMSO-treated CCI group. NAD, nicotinamide adenine dinucleotide; CCI, chronic constriction injury; H4-k16Ac, H4-k16 acetylation; DMSO, dimethyl sulfoxide.

**Figure 7:**
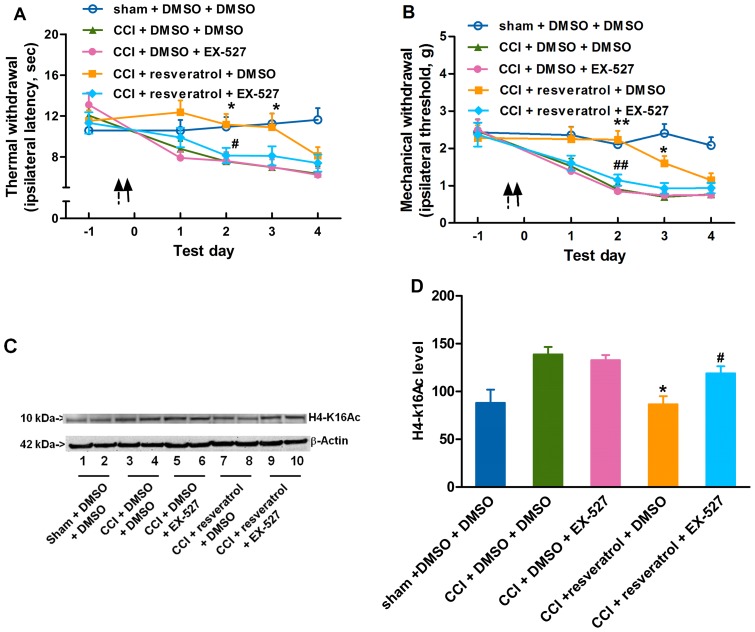
EX-527 inhibits the anti-nociceptive effect of resveratrol. Intrathecal injection of 5 µl EX-527 (1.2 mM) 1 h before resveratrol administration effectively reverses the effect of resveratrol (45 mM) delaying the onset of (A) thermal hyperalgesia and (B) mechanical allodynia produced by CCI surgery. (C) EX-527 inhibits the effect of resveratrol attenuating CCI-induced elevation of H4-K16Ac 1 day after CCI surgery. (D) Quantification of the immunoblots shows that EX-527 inhibits the effects of resveratrol on H4-K16Ac. An arrow indicates a resveratrol injection and a dotted arrow indicates an EX-527 injection. *n* = 10 in each group for behavior test and *n* = 6 in each group for H4-K16Ac analysis; ^*^ *P*<0.05, ^**^ *P*<0.01 *vs.* DMSO-DMSO-treated CCI group; ^#^ *P*<0.05, ^##^ *P*<0.01 *vs.* resveratrol-DMSO-treated CCI group. CCI, chronic constriction injury; H4-k16Ac, H4-k16 acetylation; DMSO, dimethyl sulfoxide.

To confirm that SIRT1 was a key factor through which NAD or resveratrol attenuated the development of neuropathic pain, we further observed the effect of SIRT1 inhibitor EX-527 on the anti-nociceptive effect of NAD or resveratrol. It was found that an intrathecal injection of 5 µl EX-527 (1.2 mM) [24] 1 h before NAD administration effectively blocked the anti-nociceptive effect of 800 mM NAD as compared with the DMSO-treated NAD-CCI group (Fig. 6). Additionally, an intrathecal injection of 5 µl EX-527 (1.2 mM) 1 h before resveratrol administration also effectively blocked the anti-nociceptive effect of 90 mM resveratrol as compared with the DMSO-treated resveratrol-CCI group (Fig. 7). These results indicated that SIRT1 may be the major effector of the exogenous NAD or resveratrol that prevented the development of neuropathic pain induced by CCI. Furthermore, the requirement of SIRT1 about 24 h prior to CCI surgery for NAD and resveratrol and SIRT1's predominant nuclear location [39] suggested that SIRT1 regulated a genetic program of the anti-nociceptive effect of NAD or resveratrol in the CCI-induced neuropathic pain.

## Discussion

The present study has demonstrated that the SIRT1 content/activity and NAD/NAM ratio in the spinal cord were decreased in CCI mice. Additionally, the elevation of spinal SIRT1 deacetylase activity after the intrathecal administration of NAD or resveratrol transiently inhibited the onset of CCI-induced thermal hyperalgesia and mechanical allodynia in mice. Finally, we found that an intrathecal injection of SIRT1 inhibitor EX-527 before NAD or resveratrol administration reversed the anti-nociceptive effect of NAD or resveratrol in CCI mice. Nevertheless, these findings only represent our pilot investigation and preliminary assumption that SIRT1 and NAD may be involved in neuropathic pain. The long-term goal is to further determine the most effective modulated way of SIRT1 in attenuating neuropathic pain and the underlying mechanism.

SIRT1 may play some role in an epigenetic mechanism underlying CCI-induced neuropathic pain. The anatomical analysis of SIRT1 immunoreactivity highlights the presence of SIRT1 in all four major divisions of both adult rodent and human spinal cord including the cervical, thoracic, lumbar and sacral columns [39]. SIRT1 has been implicated in molecular pathways associated with pain [40] and nerve injury [18]. CCI produced by sciatic nerve ligature [32] enables nerve cells to mobilize a reprogramming process, which involves a variety of alternations in gene modification or gene mRNA expression [4], [41]. It was found in this study that the SIRT1 expression and activity were decreased in CCI mice (Fig. 1 and 3) and modulations of SIRT1 activity by NAD (Fig. 4) or resveratrol (Fig. 5) ameliorated the development of neuropathic pain, suggesting that SIRT1 and its substrates [42] may be involved in the reprogramming process of the development of neuropathic pain. However, further studies are needed to observe pain behavioral changes in spinal SIRT1 (−/−) mice so as to clarify the direct contributions of SIRT1 to pain.

The decline in NAD and the resultant loss of genome silencing cause alternations in the related gene expression [43]. We demonstrated that NAD was decreased after CCI surgery (Fig. 2) and the exogenous NAD could ameliorate the development of neuropathic pain induced by CCI surgery (Fig. 4), suggesting that the pain neural circuit in the spinal cord may be susceptible to the NAD aberration. To further clarify the direct effect of reduced NAD on hyperalgesia, further study in nicotinamide nucleotide adenylyltransferase 1 (Nmnat1) (−/−) mice may be helpful. It was reported [44] that the calorie restriction (CR) treatment increased the NAD/NAM ratio in the brain and prevented experimental amyloid neuropathology, but it was not clear whether CR could increase NAD in the spinal cord and attenuate neuropathic pain. Our ongoing study will focus on the effect of some different living habits such as CR and environmental enrichment on the NAD content and neuropathic pain in mice.

Knowing that resveratrol has poor bioavailability when given systemically such as via oral administration [45], we administered resveratrol intrathecally and observed its local pharmacology in the spinal cord. Some studies [46], [47] reported that resveratrol was not a direct activator of SIRT1 in neuroprotection. Although resveratrol had multiple effects on many mammalian enzymes which complicated the interpretation of its effects, many effects of resveratrol in mice were consistent with the activation of SIRT1. Resveratrol was reported to extend lifespan in invertebrate organisms through activating SIRT1 homologues [37]. Specifically, resveratrol had beneficial effects in mice, which depended on SIRT1 [38]. In addition, these studies were carried out in *vitro*, and therefore it was possible that resveratrol might activate SIRT1 through some intermediate factors. Our *vivo* study showed that EX-527 as a SIRT1 inhibitor could reverse the effect of resveratrol, suggesting that the anti-nociceptive effect of resveratrol may be due to, at least in part, resveratrol-induced SIRT1 activation. However, it could not be excluded that resveratrol might attenuate the elevation of H4-k16Ac induced by CCI surgery through other pathways other than the SIRT1 pathway. To further clarify whether SIRT1 activation could attenuate neuropathic pain, our ongoing study will focus on the effect of other specific SIRT1 activators on CCI mice. The effects of NAD and resveratrol were delayed almost 2 days by paradigm pretreatment (Fig. 4, 5), suggesting that the drug effect and epigenetic modification may be transient and reversible if the drugs are given for only one or two times, indicating that continual intrathecal injections of NAD or resveratrol are needed to achieve a long-lasting anti-hyperalgesic effect. Whether the post-treatment of NAD or resveratrol could inhibit the established CCI-induced pain also needs further study. Knowing that SIRT1 is a NAD-dependent protein deacetylase and NAD is only a co-factor in SIRT1's deacetylation, a single or two administrations of NAD may not be able to produce long-lasting effects. Additionally, our data showed that the intrathecal injections of NAD were done 2 times at 24 h intervals while resveratrol was injected once and showed longer and stronger anti-hyperalgesic effects. There may be two underlying reasons. Firstly, NAD was quickly consumed to NAM by SIRT1, while resveratrol activated SIRT1 for a longer time. Secondly, resveratrol was more hydrophobic than NAD, and was easier to go through the cell membrane and then reach the target site, showing stronger action than NAD. Both of the above presumptions need to be further studied. A most recent study [21] reported that the SIRT1 content was decreased after CCI surgery in rats, and this decrease was consistent with the behavioral change. However, they did not investigate the effect of the increased SIRT1 activity on attenuating CCI-induced neuropathic pain, and therefore their finding could not strongly support that CCI-induced hyperalgeisa was attributed to the reduction of SIRT1. In addition, their study did not show the effect of sham surgery on SIRT1 content and activity. Our study demonstrated that SIRT1 content and activity were decreased in CCI mice, but not in sham surgery mice (Fig. 1 and 3). We also found that the elevation of SIRT1 activity by exogenous NAD or resveratrol ameliorated the development of neuropathic pain induced by CCI surgery and SIRT1 inhibitor EX-527 reversed the anti-nociceptive effect of NAD or resveratrol, suggesting that SIRT1 may be a key factor contributing to CCI-induced neuropathic pain. Our future study will investigate the down-stream mechanism through which SIRT1 attenuates neuropathic pain.

In conclusion, our in *vivo* results have provided some evidence that SIRT1 plays a role in ameliorating the development of neuropathic pain, which may be a novel mechanism underlying neuropathic pain. It may be possible that the manipulation of SIRT1 activity by NAD and/or resveratrol in epidural anaesthesia combined with local anesthetics could provide new therapeutic opportunities for the treatment of neuropathic pain induced by nerve injuries associated with some surgical procedures such as amputation or thoracic operation.
